# Key Considerations for Frail Patients Undergoing Hip Fracture Surgery

**DOI:** 10.3390/clinpract14060177

**Published:** 2024-10-23

**Authors:** Ana-Maria Dumitriu, Rǎzvan Ene, Liliana Mirea

**Affiliations:** 1 Faculty of Medicine, ”Carol-Davila” University of Medicine and Pharmacy, 020021 Bucharest, Romania; llmirea@yahoo.com; 2 Anaesthesiology and Intensive Care Clinic, Clinical Emergency Hospital Bucharest, 105402 Bucharest, Romania; 3 Orthopedics and Trauma Surgery, Clinical Emergency Hospital Bucharest, 105402 Bucharest, Romania

**Keywords:** elderly, frailty, hip fracture, risk stratification, multidisciplinary team, optimization, preoperative, co-morbidities, timely surgery

## Abstract

Improving preoperative care for elderly patients with hip fractures is crucial for achieving the best outcomes. A multidisciplinary team that can improve overall care quality by addressing patient’s medical conditions, analgesia, timely surgery, and early postoperative mobilization is required. This narrative review provides insights regarding the extent of preoperative optimization needed for hip fracture surgery.

## 1. Introduction

Hip fractures secondary to falls represent a major global healthcare burden. Approximately 400,000 new cases were recently registered in the central European region, resulting in 55,000 cases with Years Lived with Disability attributable to hip fractures, with nearly 10% of the cases recorded in Romania [1]. Current data still suggest a strong association between age and the risk of hip fracture, with a decreasing tendency in females and an increasing tendency in males [2]. Hip fractures are associated with devastating complications, such as respiratory, cardiac, and multiorgan failures, and overall, increase baseline mortality risk [3]. Up to 60% of patients are confronted with long-term disability, impaired functionality, and low quality of life [4].

This narrative review seeks to highlight the relevant preoperative interventions amongst patients with hip fractures and significant medical conditions.

Surgical treatment remains the main treatment option for most hip fractures. Conservative management may be considered an option in patients who are already bed immobilized, terminally ill, or have a severe neurocognitive dysfunction, as long as effective pain control is provided [5].

Some studies recommend a shorter time to surgical treatment, in a 12 h therapeutic window [6,7], while Pincus D. et al. concluded that waiting time should not exceed 24 h [8].

In the context of this controversy, the most current large-scale randomized controlled trial, HIP ATTACK (Accelerated surgery versus standard care for hip fractures) aimed to shed light on the issue. The study outlined that a 6 h window to surgery has similar outcomes as standard care surgery performed within 24 h [9].

On the other hand, we must not overlook the fact that there are authors who believe that if a correctable medical condition is identified and optimization is possible, surgical delay may be justified up to 48 h after the event [10].

We firmly believe that intentionally postponing surgery without valid medical reasons is unethical. Surgical delays may be unavoidable in patients deemed unfit for surgery at the point of admission due to compromised health status or a serious decompensated chronic condition. In this scenario, it is essential to recognize that hasty surgical decisions might limit opportunities for optimizing patients’ medical conditions. Nevertheless, we believe that there are an extremely limited number of clinical scenarios that would justify delaying surgical intervention to or beyond 48 h.

## 2. Pain Control in Acute Settings

Traumatic pain in geriatric patients can be underrated, particularly in those with ongoing neurodegenerative disease. Intracapsular fractures are considered to be less painful than extracapsular injuries, which are accompanied by a more severe periosteal injury [11].

**Figure 1:**
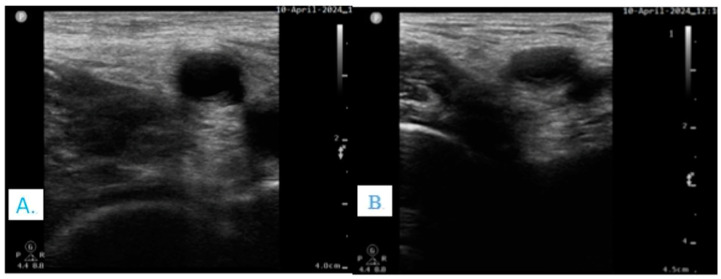
US image of femoral nerve: (**A**) Normal finding. (**B**) Patient with a hip fracture and hypovolemia: due to local edema, the femoral nerve tends to dive underneath the femoral artery. (*Authors personal archive*).

**Figure 2:**
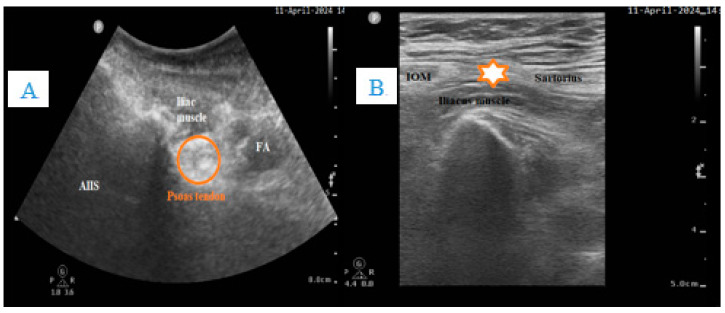
(**A**). US image of PENG block. (**B**) US image of supra-inguinal fascia iliaca block. (*Authors personal archive*). AIIS: anterior inferior iliac spine, FA: Femoral artery, IOM: internal oblique muscle.

Pain management should start immediately in the emergency department and our target should be to diminish the requirement for pharmacologic interventions [12]. In this regard, peripheral nerve blocks are well-tolerated opioid-sparring techniques that provide superior analgesia. The choice of nerve block depends on patient consent, particularities (e.g., anatomy, chronic antiplatelet or anticoagulant treatment), and clinicians’ experience and skills. An ultrasound (US)-guided single shot femoral nerve block (Figure 1) is a safe and effective intervention for pain relief that may be performed immediately after diagnosis [13]. Newer techniques such as ultrasound-guided supra-inguinal fascia iliaca or pericapsular nerve group (PENG) blocks (Figure 2) are also excellent alternatives [14,15].

Systemic analgesia still plays an important role in the pain control strategy. Current literature supports acetaminophen as the first choice [16]. Nonsteroidal Anti-Inflammatory Drugs (NSAIDs) play an important role in controlling the inflammatory response, but usage should be limited due to their adverse renal effects [17,18]. Opioid analgesia should not be regularly administered [5]. Gabapentin does not offer better pain control in patients undergoing orthopedic surgery [19].

## 3. Stratifying the Risk in Frail Patients

A preoperative evaluation is crucial for patients with hip fractures to clarify the mechanism of injury, detect a possible underlying condition that led to the fall, and identify an acute medical condition as well as additional injuries (such as other fractures or even subdural hematoma/hemorrhage). The assessment may often be challenging, given that a significant percentage of patients with hip fractures have at least moderate cognitive impairment; therefore, supplementary medical information needs to be obtained from relatives and/or caregivers.

We discuss below some of the most important medical conditions that require prompt intervention.

### 3.1. Anemia

Anemia is a common finding in patients presenting to the emergency department with hip fractures. In 50% of cases, acute or chronic anemia is detected in the preoperative settings and up to 90% of patients acquire postoperative anemia, with an average hemoglobin drop of 25 g/L [20,21]. Anemia correction is an essential step to recovering functional independence and early mobilization, although a strong consensus on the optimal transfusion threshold has not been defined yet. However, transfusion should not delay surgery. A Cochrane systematic review concluded that a restrictive transfusion strategy has no significant impact on mortality or complication rates [22]. A more liberal transfusion strategy may be advisable for patients with significant pre-existing cardiac disease, although less can sometimes be more [23,24]. Consequently, a threshold of 8 g/dL is recommended for most orthopedic surgical patients (13). In order to reduce blood transfusion in patients with hip fractures, recent data support the preoperative use of IV iron and tranexamic acid, combined or alone [25]. Anemia is usually worse following the intramedullary nail and dynamic hip screw surgery technique [21].

### 3.2. Thrombocytopenia

Thrombocytopenia, defined by a platelet count of less than 50 to 80 × 10^9^/L, usually requires a hematologist’s advice, a blood film, and an evaluation of antiplatelet medication [21]. A platelet transfusion is mandatory before surgery.

### 3.3. Volume Depletion

In patients with hip fractures, volume depletion is either due to bleeding at the fracture site or, more often, a result of poor nutrition, inappropriate water intake, and polypragmasia [26,27]. In some cases, a prolonged collapse secondary to a fall may lead to rhabdomyolysis and precipitate acute kidney injury [28].

Fluid imbalance requires intravenous fluid resuscitation and maintenance under the careful guidance of clinical signs and point-of-care US parameters [29,30]. Both under-resuscitation and excessive fluid resuscitation have a negative impact on patient outcomes [31,32]. Since colloids failed to demonstrate additional benefits, the prudent use of balanced crystalloids is preferred to maintain tissue perfusion [26]. It is advisable to avoid 0.9% sodium chloride, especially in large volumes, as it can be a source of dilutional hyperchloremic metabolic acidosis and other unwanted consequences [33].

However, hypovolemia and electrolyte imbalance are frequently complementary. Profound plasma electrolyte abnormalities like severe hypo or hypernatremia and hypo or hyperkalemia postpone surgery care and demand preoperative correction [26].

In patients with fragility fractures, hyponatremia is rather multi-factorial (secondary to dehydration, chronic medication, and syndrome of inappropriate antidiuretic hormone (SIADH) secretion) [34]. Severity, rate of onset, and fluid status must be evaluated. Even though the predominant concern is to avoid rapid overcorrection of serum sodium levels, we must bear in mind that slow correction (less than 6 mEq/l/24 h) is also detrimental [35]. Despite the traditional association between rapid sodium correction and central pontine myelinolysis, recent research supports a sodium correction rate of more than 8–10 mEq/l/24 h [35]. In the absence of large prospective trials with adjustments for co-morbidities and disease-specific guidance, we consider that the sodium correction rate should not exceed more than 8–10 mEq/l/24 h. Regular blood tests are preferred to formulas to check an improving trend.

**Hypernatremia** is most commonly due to dehydration and is less frequently a result of iatrogenic infusion of hypertonic solutions. Intravenous fluid should be administered with caution to prevent rapid shifts in sodium plasma levels. In the presence of hypernatremia, a hyperosmolar, hyperglycemic state should be presumed; therefore, blood glucose levels and serum osmolality checks have to be considered (especially in patients with type 2 diabetes) [36].

A retrospective cohort analysis by Klinck et al. demonstrated a harmful effect when sodium levels either increased or decreased by more than 5 mmol/L preoperatively [31].

**Hypokalemia** and **hyperkalemia** have similar prevalences at the time of admission. Although both are linked to possibly lethal cardiac arrhythmias, **hypokalemia** is associated with a worse outcome [32]. The possible causes of hypokalemia need to be considered (e.g., diuretics or gastrointestinal losses). In emergency settings, mild hypokalemia is treated with intravenous therapy and additional potassium chloride. If plasma K^+^ levels are not lower than 3 mmol/L, preparation for surgery may proceed as long as replacement is in progress and no relevant ECG changes are present [21]. We must take into account that hypomagnesemia is closely correlated with hypokalemia, thus plasma magnesium levels should also be checked and corrected if required. **Hyperkalemia** is usually a result of potassium-sparing diuretics and/or angiotensin-converting enzyme inhibitor medications, dehydration, or acute kidney failure. Unless severe hyperkalemia is encountered, conservative treatment (such as insulin/dextrose infusions) should be started and surgical preparation may continue [21].

### 3.4. Cardiovascular Events

Cardiovascular events, such as myocardial ischemia or cardiac failure, tend to be more frequent preoperatively [37]. All patients should be screened for potential related risk factors, new signs or symptoms, or established cardiovascular disease. A 12-lead ECG and measurement of troponin level are recommended before surgery [38]. BNP or NT-proBNP can also be considered [39]. In addition, risk-prediction tools such as RCRI (Revised Cardiac Index), ACS NSQIP (The American College of Surgery National Surgical Quality Improvement Program), SORT (Surgical Outcome Risk Tool), or AUB (The American University of Beirut) HAS2 Cardiovascular Risk Index may be used, although no risk calculator is perfect and thus cannot replace clinical examination or judgment [40]. A focused cardiac US (FOCUS) may be used complementarily with auscultation, although its accuracy in evaluating structural and functional deficiencies is limited [41].

Patients with poor functional capacity, elevated biomarker levels, new signs and symptoms (e.g., murmur, chest pain, dyspnea, or peripheral edema), or abnormal ECG may benefit from preoperative cardiac assessment [38]. The presence of an implanted cardiac pacemaker or cardioverter defibrillator requires pacemaker interrogation and often requires a mode compatible with diathermy [21].

Supraventricular arrhythmias with a heart rate of more than 120 beats/min require ventricular rate control before proceeding to surgery [26]. If a second or third-degree atrioventricular block is identified, temporary pacemaker placement may be necessary [5].

The decision to perform supplementary and potentially unnecessary checkups (e.g., routinely transthoracic echocardiography) in the absence of a high-risk cardiovascular profile may aggravate the patient’s prognosis [42,43]. In a time-sensitive scenario, a supplementary delay of 9 h (as shown by Smeets et al.), 24 h (as demonstrated by Bellas et al.), or even 6 days (as found by Mutlu et al.) for cardiac consultation is not justified [44,45,46].

### 3.5. Treatment and Bleeding Risk

Hip fracture surgery carries a significant risk of bleeding and, for up to 40% of these patients, chronic use of anticoagulant and/or antiplatelet medications increases the risk substantially [16]. Our everyday practice dilemma is to balance four major aspects: surgical bleeding risk, a possible contraindication for regional anesthesia, immediate cessation of treatment, and surgery postponement.

#### 3.5.1. Antiplatelet Medication

Routinely, the withdrawal of **antiplatelet medication** prior to surgery is not advisable. The risk of severe cardiovascular complications and even acute stroke must be taken into account, especially in patients with an important cardiovascular history [5].

Antiplatelet monotherapy is usually not withheld [16]. In the case of dual antiplatelet therapy, it is mandatory to establish the reason for this treatment option. Although specific guidance is not available, surgery should not be delayed and the type of anesthesia should be discussed on a case-by-case basis [16]. Point-of-care measurement of platelet inhibition may be considered [47].

The decision to withhold the P2Y12 inhibitor should be based on how much time has passed since an acute coronary syndrome event or coronary stenting procedure [5].

The decision to stop clopidogrel is rather conflicting. There is strong evidence suggesting that clopidogrel should not be stopped [48]. However, Tarrant et al. suggested that both the continuation of dual antiplatelet medication before hip fracture repair and delays to surgery for those on this treatment were associated with higher mortality rates [49,50].

#### 3.5.2. Anticoagulation Therapy

**Anticoagulation therapy** is a frequent reason for postponing surgery, but time off treatment should generally be reduced as much as possible [51].

The risk of withholding **warfarin** therapy varies according to the indication and prevalent pro-thrombotic predisposition [21]. Preoperative INR levels must be checked. Surgical treatment is safe if INR is less than 1.8 and neuraxial anesthesia may be considered if INR is less than 1.5 [16,52]. Warfarin therapy should be reversed depending on the associated comorbidities (e.g., uncomplicated atrial fibrillation, deep vein thrombosis) and INR levels. Administering intravenous vitamin K or prothrombin concentrate complex may be necessary [16]. Bridging therapy is indicated for patients with high thrombotic risk conditions such as mechanical mitral or aortic valves, atrial fibrillation CHA2DS2–VASc scores of 7–9, history of stroke, venous thromboembolism within 3 months, or severe thrombophilia [51].

**Direct oral anticoagulant drugs (DOACs)** require a different approach due to their rapid onset of action and variable peak plasma concentrations following administration (starting from one hour and a half to four hours) [21].

Dabigatran has the highest renal removal rate (80%), whereas edoxaban, rivaroxaban, and apixaban have lower rates (50%, 35%, and 25–27%, respectively) [53]. Clotting function tests are not reliable for drug activity, although PT and INR may be prolonged [16]. Thrombin time (TT) successfully detects the activities of direct thrombin inhibitors such as dabigatran [54]. If the TT level is normal, we can proceed with surgery and anesthesia (including spinal, if indicated); if prolonged, we can consider reversal with a specific antidote [52,55].

In the absence of specific recommendations for the management of DOACs during emergency surgery, waiting two half-lives between the last dose and surgery offers an appropriate compromise between risk and benefit [52]. Knowing that the half-life of DOACs is up to 15 h and their clearance is markedly dependent on renal function, it is advisable to schedule hip surgery for the day after admission [23]. However, some authors claim that a 24 h postponement might have a negligible effect on reversing DOACs’ effects [52].

A test for the specific DOAC level should be performed on the morning of the surgery only if creatinine clearance is at least 30 mL/min^−1^ [16]. If the DOAC level is ≤50 ng/mL, surgery may proceed [16,21]. If a DOAC-specific assay is not available, patients with creatinine clearance < 30 mL/min^−1^ should be scheduled for surgery four half-lives (48 h) after the last dose (spinal anesthesia may be administered) [16].

In selected cases, an individual risk–benefit evaluation and a multidisciplinary approach outside the novel guidelines may be required [56].

Oral anticoagulation may be resumed 24–48 h postoperatively in the absence of active bleeding or severe anemia [52].

### 3.6. Diabetic Patients

Diabetic patients with hip fractures are often prone to unpredictable glycemic control, which considerably contributes to further complications [57].

Blood glucose levels can be markedly elevated due to stress response or unacceptably low due to preoperative fasting or inadequate antidiabetic treatment. **Hyperglycemia** may be seen in up to 47% of patients without diabetes as a normal stress response to trauma, with even higher levels postoperatively [58,59].

A perioperative serum glucose level of 140–180 mg/dL is our target. Serum glucose levels higher than 180 mg/dL may require basal insulin (0.1–0.15 U/kg) as well as correctional insulin treatment [16]. A more intensive treatment is required for ketoacidosis [60].

Surgery care may proceed if the glucose level is less than 360 mg/dL and on a downward trend. Insulin-dependent patients should be scheduled first on the surgical timetable [60]. Unless glycemic control is poor, preoperative fasting should be limited to 6 h for solid foods and 2 h for clear liquids before surgery care [60]. Blood glucose levels should be checked closely during the entire perioperative period and enteral nutrition resumed as soon as possible following surgery [16].

### 3.7. Extensive Pulmonary Assessment

Apart from clinical evaluation and information about risk factors, extensive **pulmonary assessment** is usually reserved for patients with chronic conditions such as chronic obstructive pulmonary disease, asthma, and obstructive sleep apnea [61]. Chest X-rays and functional tests such as spirometry or arterial blood gas analysis are not routinely required in asymptomatic patients [61,62]. The presence of acute exacerbation and hypoxemia require further evaluation and may represent a solid reason for surgical delay [5,61].

Delaying surgery for hip fractures in patients with preoperative pneumonia has no benefits [63]. However, this finding is not irrevocable and an individual approach on a case-by-case basis may be wiser. A recent analysis concluded that preoperative pneumonia significantly increased complication rates in patients undergoing elective surgery [64].

### 3.8. Chronic Kidney Disease

Chronic kidney disease is frequently encountered in the geriatric population and acute kidney injury (AKI) is reported in 24% of cases [28]. Serum creatinine level is not a reliable marker and renal dysfunction may be present even when serum creatinine levels are within the usual range [28]. In consequence, creatinine clearance (Cockcroft Gault formula) may be more adequate [65]. Novel biomarkers, such as plasma or urinary NGAL (neutrophil gelatinase-associated lipocalin) or plasma proenkephalin-A concentration, can be used for the prediction of AKI, if available [66,67]. However, serum creatinine levels, as well as certain drugs (ACEis, ARBs, NSAIDs), blood transfusion, and history of cardiovascular disease, increase the risk of AKI [22,68]. For these patients, certain medications and fluid balance need regular adjustments to minimize the occurrence of unwanted complications [22].

### 3.9. Infection

Infection should be suspected when the white blood cell count is more than 17 × 10^9^/L, while lower levels can usually accompany a traumatic inflammatory response [26]. The most frequent infections encountered in preoperative settings are urinary tract and upper/lower tract respiratory infections [50,69]. Asymptomatic bacteriuria is also common and sometimes difficult to distinguish from a urinary tract infection [70]. Preoperative urinary tract infections expose patients to a higher risk of inferior functional outcomes; however, early diagnosis of symptomatic UTIs and appropriate antibiotic therapy may improve the clinical course [71]. Prophylactic antibiotics administered up to 2 h before surgery can reduce postoperative complications such as wound infection, urinary tract infection, and respiratory infection [72]. Except for sepsis or septic shock, infections generally should not delay surgery care [16].

### 3.10. Chronic Medication

Abrupt cessation of beta blockers, Parkinson’s disease medication, levothyroxine, and corticosteroids may lead to unwanted complications and should usually be continued perioperatively [73].

Several other drugs such as angiotensin-converting enzyme inhibitors (ACEis) and angiotensin II receptor antagonists (ARBs), oral antidiabetic therapies, diuretics, and NSAIDs should be discontinued in the preoperative period [73].

Special care is required for patients on psychotropic medication since some classes are usually safe to use in the perioperative period (e.g., haloperidol, risperidone, olanzapine), while lithium and type B monoamine oxidase inhibitors should be stopped [21,74].

### 3.11. Cognitive Shifts

Cognitive shifts, such as delirium, do not have a specific timeframe and may be present upon admission and sometimes mask an ongoing neurocognitive disorder or arise in every other perioperative stage [75]. Time to surgery, chronic conditions, medical therapy (especially anticholinergics and narcotics), as well as unfamiliar surroundings, can lead to preoperative delirium [76]. Pain control through peripheral nerve blocks is one of the most valuable strategies for decreasing the incidence of perioperative cognitive disorders [77]. Treatment should focus on non-pharmacological options whenever possible, for example, reverse contributing factors, avoid further complications (e.g., dehydration), and provide psychological and functional support [5]. Medical intervention should be used only when the aforementioned strategies have failed or the patient may harm him/herself or others and not prophylactic [76].

## 4. Other Preoperative Considerations

More than half of hip fractures in the elderly occur intracapsular and the vast majority of these patients undergo hemiarthroplasty. The controversy related to the stability of cemented or uncemented prostheses is ongoing. A recent meta-analysis showed that cemented implants allow faster recovery and lower pain scores compared to non-cemented prostheses [77]. Unfortunately, the process of cementing the femoral canal is still accompanied by severe side effects and even death.

Compared with elective arthroplasties, bone cement implantation syndrome generates intraoperative complications six times more frequently in patients with hip fractures, and mortality is even higher.

Associated cardiopulmonary pathologies are factors involved in the systemic inflammatory response along with acidosis and hypoxemia. At the same time, certain preoperative medications are also incriminated, including diuretics, beta-blockers, DOACs, and ACEis. Appropriate fluid resuscitation in addition to 5-hydroxytryptamine (5-HT3) receptor antagonists, histamine receptor blockers, and corticosteroids should be considered in patients undergoing cemented hip arthroplasty to reduce the risk of bone cement implantation syndrome [78]. The presence of polymethyl methacrylate monomer (PMMA) in the composition of cement may cause important vascular muscular relaxation; however, this hypothesis has not been demonstrated [79]. An embolus-mediated model, complement activation, and histamine release have been proposed as physio-pathological mechanisms [79]. An uncemented prosthetic component can be used right from the start, but this is concretely related to the quality of the bone [80]. Equally, low-viscosity cement can be used to reduce the pressure in the femoral canal during cementation, preferably through the vacuum technique, to reduce the porosity of the cement [81].

In order to avoid these intraoperative complications, close collaboration between surgeon and anesthetist and rigorous preoperative planning are essential.

## 5. Conclusions

An intensive approach to preoperative care for patients with hip fractures may improve outcomes and reduce perioperative complications. Addressing comorbidities, using peripheral nerve blocks to minimize opioid dependency, prompt surgical repair, and early mobilization are key concepts for enhancing recovery pathways and preventing organ failure. It is imperative to recognize the time-sensitivity of hip fractures and ensure that a multidisciplinary team is involved in providing comprehensive care. Multidisciplinary teams can facilitate better communication among team members, leading to more coordinated and efficient care delivery.

## References

[1] Ilic I., Ristic B., Stojadinovic I., Ilic M. (2023). Epidemiology of Hip Fractures due to Falls. Medicina.

[2] Vos T., Lim S.S., Abbafati C., Abbas K.M., Abbasi M., Abbasifard M., Abbasi-Kangevari M., Abbastabar H., Abd-Allah F., Abdelalim A. (2020). Global burden of 369 diseases and injuries in 204 countries and territories, 1990–2019: A systematic analysis for the Global Burden of Disease Study 2019. Lancet.

[3] Groff H., Kheir M.M., George J., Azboy I., Higuera C.A., Parvizi J. (2019). Causes of in-hospital mortality after hip fractures in the elderly. HIP Int..

[4] Dyer S.M., Crotty M., Fairhall N., Magaziner J., Beaupre L.A., Cameron I.D., Sherrington C., Fragility Fracture Network (FFN) Rehabilitation Research Special Interest Group (2016). A critical review of the long-term disability outcomes following hip fracture. BMC Geriatr..

[5] Ackermann L., Schwenk E.S., Lev Y., Weitz H. (2021). Update on medical management of acute hip fracture. Clevel. Clin. J. Med..

[6] Bretherton C.P., Parker M.J. (2015). Early surgery for patients with a fracture of the hip decreases 30-day mortality. Bone Jt. J..

[7] Uzoigwe C.E., Burnand H.G.F., Cheesman C.L., Aghedo D.O., Faizi M., Middleton R.G. (2013). Early and ultra-early surgery in hip fracture patients improves survival. Injury.

[8] Pincus D., Ravi B., Wasserstein D., Huang A., Paterson J.M., Nathens A.B., Kreder H.J., Jenkinson R.J., Wodchis W.P. (2017). Association Between Wait Time and 30-Day Mortality in Adults Undergoing Hip Fracture Surgery. JAMA.

[9] Lizaur-Utrilla A., Lopez-Prats F.A. (2020). Hip attack for hip fractures: Is ultra-early surgery necessary?. Lancet.

[10] Seong Y.J., Shin W.C., Moon N.H., Suh K.T. (2020). Timing of Hip-fracture Surgery in Elderly Patients: Literature Review and Recommendations. Hip Pelvis.

[11] Dixon J., Ashton F., Baker P., Charlton K., Bates C., Eardley W. (2018). Assessment and Early Management of Pain in Hip Fractures: The Impact of Paracetamol. Geriatr. Orthop. Surg. Rehabil..

[12] Overview|Hip Fracture: Management|Guidance|NICE. https://www.nice.org.uk/guidance/cg124.

[13] Ketelaars R., Stollman J.T., van Eeten E., Eikendal T., Bruhn J., van Geffen G.-J. (2018). Emergency physician-performed ultrasound-guided nerve blocks in proximal femoral fractures provide safe and effective pain relief: A prospective observational study in The Netherlands. Int. J. Emerg. Med..

[14] Kelly T., Moore B., George R. (2024). Improving morbidity and mortality in hip fragility fractures. Curr. Opin. Anaesthesiol..

[15] Chen L., Shen Y., Liu S., Cao Y., Zhu Z. (2021). Ultrasound-guided supra-inguinal fascia Iliaca compartment block for older adults admitted to the emergency department with hip fracture: A randomized controlled, double-blind clinical trial. BMC Geriatr..

[16] Griffiths R., Babu S., Dixon P., Freeman N., Hurford D., Kelleher E., Moppett I., Ray D., Sahota O., Shields M. (2021). Guideline for the management of hip fractures 2020: Guideline by the Association of Anaesthetists. Anaesthesia.

[17] Pavelescu D., Mirea L., Păduraru M., Beuran M., Chiotoroiu A., Grinţescu I. (2011). The role of multimodal analgesia in the decrease of postoperative surgical stress response in major neoplastic thoraco-abdominal surgery. Chirurgia.

[18] Zhang X., Donnan P.T., Bell S., Guthrie B. (2017). Non-steroidal anti-inflammatory drug induced acute kidney injury in the community dwelling general population and people with chronic kidney disease: Systematic review and meta-analysis. BMC Nephrol..

[19] Eloy J.D., Anthony C., Amin S., Caparó M., Reilly M.C., Shulman S. (2017). Gabapentin Does Not Appear to Improve Postoperative Pain and Sleep Patterns in Patients Who Concomitantly Receive Regional Anesthesia for Lower Extremity Orthopedic Surgery: A Randomized Control Trial. Pain Res. Manag..

[20] Spahn D.R. (2010). Anemia and Patient Blood Management in Hip and Knee Surgery: A Systematic Review of the Literature. Anesthesiology.

[21] Parke S., Eaves C., Dimond S., Bainbridge C., Bellwood J., Mattinson D. (2020). Perioperative optimization of hip fracture patients. Orthop. Trauma.

[22] Guay J., Parker M.J., Gajendragadkar P.R., Kopp S. (2016). Anaesthesia for hip fracture surgery in adults. Cochrane Database Syst. Rev..

[23] Gu W.J., Gu X.P., Wu X.D., Chen H., Kwong J.S.W., Zhou L.Y., Chen S., Ma Z.-L. (2018). Restrictive versus liberal strategy for red blood-cell transfusion: A systematic review and meta-analysis in orthopaedic patients. J. Bone Jt. Surg..

[24] Ștefan M., Tomescu D., Predoi C., Goicea R., Perescu M., Popescu M., Dorobanțu D., Droc G., Andrei Ș., Știru O. (2023). Less (Transfusion) Is More—Enhancing Recovery through Implementation of Patient Blood Management in Cardiac Surgery: A Retrospective, Single-Centre Study of 1174 Patients. J. Cardiovasc. Dev. Dis..

[25] Lasocki S., Capdevila X., Vielle B., Bijok B., Lahlou-Casulli M., Collange V., Grillot N., Deserts M.D.D., Duchalais A., Delannoy B. (2023). Ferric derisomaltose and tranexamic acid, combined or alone, for reducing blood transfusion in patients with hip fracture (the HiFIT trial): A multicentre, 2 × 2 factorial, randomised, double-blind, controlled trial. Lancet Haematol..

[26] Griffiths R., Alper J., Beckingsale A., Goldhill D., Heyburn G., Holloway J., Leaper E., Parker M., Ridgway S., White S. (2012). Management of proximal femoral fractures 2011: Association of Anaesthetists of Great Britain and Ireland. Anaesthesia.

[27] Kumar D., Mbako A.N., Riddick A., Patil S., Williams P. (2011). On admission haemoglobin in patients with hip fracture. Injury.

[28] Porter C.J., Moppett I.K., Juurlink I., Nightingale J., Moran C.G., Devonald M.A.J. (2017). Acute and chronic kidney disease in elderly patients with hip fracture: Prevalence, risk factors and outcome with development and validation of a risk prediction model for acute kidney injury. BMC Nephrol..

[29] Parker C.W., Kolimas A.M., Kotini-Shah P. (2022). Velocity-Time Integral: A Bedside Echocardiography Technique Finding a Place in the Emergency Department. J. Emerg. Med..

[30] Di Nicolò P., Tavazzi G., Nannoni L., Corradi F. (2023). Inferior Vena Cava Ultrasonography for Volume Status Evaluation: An Intriguing Promise Never Fulfilled. J. Clin. Med..

[31] Klinck J., McNeill L., Di Angelantonio E., Menon D.K. (2015). Predictors and outcome impact of perioperative serum sodium changes in a high-risk population. Br. J. Anaesth..

[32] Norring-Agerskov D., Madsen C.M., Abrahamsen B., Riis T., Pedersen O.B., Jørgensen N.R., Bathum L., Lauritzen J.B., Jørgensen H.L. (2017). Hyperkalemia is Associated with Increased 30-Day Mortality in Hip Fracture Patients. Calcif. Tissue Int..

[33] Lobo D.N., Awad S. (2014). Should chloride-rich crystalloids remain the mainstay of fluid resuscitation to prevent ‘pre-renal’ acute kidney injury?: Con. Kidney Int..

[34] Cumming K., Hoyle G.E., Hutchison J.D., Soiza R.L. (2014). Prevalence, Incidence and Etiology of Hyponatremia in Elderly Patients with Fragility Fractures. PLoS ONE.

[35] Seethapathy H., Zhao S., Ouyang T., Passos C., Sarang A., Cheung P.W., Waikar S.S., Steele D.J., Kalim S., Allegretti A.S. (2023). Severe Hyponatremia Correction, Mortality, and Central Pontine Myelinolysis. NEJM Evid..

[36] Braun M.M., Barstow C.H., Pyzocha N.J. (2015). Diagnosis and Management of Sodium Disorders: Hyponatremia and Hypernatremia. Am. Fam. Physician.

[37] Rostagno C., Polidori G., Ceccofiglio A., Cartei A., Boccaccini A., Peris A., Rubbieri G., Civinini R., Innocenti M. (2020). Takotsubo Syndrome: Is This a Common Occurrence in Elderly Females after Hip Fracture?. J. Crit. Care Med..

[38] Halvorsen S., Mehilli J., Cassese S., Hall T.S., Abdelhamid M., Barbato E., De Hert S., de Laval I., Geisler T., Hinterbuchner L. (2022). 2022 ESC Guidelines on cardiovascular assessment and management of patients undergoing non-cardiac surgery. Eur. Heart J..

[39] Duceppe E., Patel A., Chan M.T.V., Berwanger O., Ackland G., Kavsak P.A., Rodseth R., Biccard B., Chow C.K., Borges F.K. (2020). Preoperative N-Terminal Pro-B-Type Natriuretic Peptide and Cardiovascular Events after Noncardiac Surgery: A Cohort Study. Ann. Intern. Med..

[40] Glance L.G., Faden E., Dutton R.P., Lustik S.J., Li Y., Eaton M.P., Dick A.W. (2018). Impact of the Choice of Risk Model for Identifying Low-risk Patients Using the 2014 American College of Cardiology/American Heart Association Perioperative Guidelines. Anesthesiology.

[41] Canty D.J., Heiberg J., Yang Y., Royse A.G., Margale S., Nanjappa N., Scott D.A., Maier A.B., Sessler D.I., Chuan A. (2019). One-year results of the pilot multicentre randomised trial of preoperative focused cardiac ultrasound in hip fracture surgery. Anaesth. Intensive Care.

[42] Borges F.K., Bhandari M., Guerra-Farfan E., Patel A., Sigamani A., Umer M., Tiboni M.E., Villar-Casares M.d.M., Tandon V., Tomas-Hernandez J. (2020). Accelerated surgery versus standard care in hip fracture (HIP ATTACK): An international, randomised, controlled trial. Lancet.

[43] Sawhney C., Trikha V., Janani S., Bajwa S.S., Sharma V., Khanna M. (2017). Impact of preoperative echocardiography on perioperative management in geriatric hip trauma: A retrospective observational study. Int. J. Appl. Basic Med. Res..

[44] Smeets S.J.M., Poeze M., Verbruggen J.P.A.M. (2012). Preoperative cardiac evaluation of geriatric patients with hip fracture. Injury.

[45] Bellas N., Stohler S., Staff I., Majk K., Lewis C., Davis S., Kumar M. (2020). Impact of Preoperative Specialty Consults on Hospitalist Comanagement of Hip Fracture Patients. J. Hosp. Med..

[46] Mutlu H., Bilgili F., Mutlu S., Karaman O., Cakal B., Ozkaya U. (2016). The effects of preoperative non-invasive cardiac tests on delay to surgery and subsequent mortality in elderly patients with hip fracture. J. Back Musculoskelet. Rehabil..

[47] Patti G., Nusca A., Mangiacapra F., Gatto L., D’Ambrosio A., Di Sciascio G. (2008). Point-of-care measurement of clopidogrel responsiveness predicts clinical outcome in patients undergoing percutaneous coronary intervention results of the ARMYDA-PRO (Antiplatelet therapy for Reduction of MYocardial Damage during Angioplasty-Platelet Reactivity Predicts Outcome) study. J. Am. Coll. Cardiol..

[48] Levine G.N., Bates E.R., Bittl J.A., Brindis R.G., Fihn S.D., Fleisher L.A., Granger C.B., Lange R.A., Mack M.J., Mauri L. (2016). 2016 ACC/AHA Guideline Focused Update on Duration of Dual Antiplatelet Therapy in Patients with Coronary Artery Disease: A Report of the American College of Cardiology/American Heart Association Task Force on Clinical Practice Guidelines: An Update of the 2011 ACCF/AHA/SCAI Guideline for Percutaneous Coronary Intervention, 2011 ACCF/AHA Guideline for Coronary Artery Bypass Graft Surgery, 2012 ACC/AHA/ACP/AATS/PCNA/SCAI/STS Guideline for the Diagnosis and Management of Patients with Stable Ischemic Heart Disease, 2013 ACCF/AHA Guideline for the Management of ST-Elevation Myocardial Infarction, 2014 AHA/ACC Guideline for the Management of Patients with Non-ST-Elevation Acute Coronary Syndromes, and 2014 ACC/AHA Guideline on Perioperative Cardiovascular Evaluation and Management of Patients Undergoing Noncardiac Surgery. Circulation.

[49] Tarrant S.M., Kim R.G., McGregor K.L., Palazzi K., Attia J., Balogh Z.J. (2020). Dual Antiplatelet Therapy and Surgical Timing in Geriatric Hip Fracture. J. Orthop. Trauma.

[50] Lizaur-Utrilla A., Gonzalez-Navarro B., Vizcaya-Moreno M.F., Miralles Muñoz F.A., Gonzalez-Parreño S., Lopez-Prats F.A. (2019). Reasons for delaying surgery following hip fractures and its impact on one year mortality. Int. Orthop..

[51] Doherty J.U., Ty Gluckman C.J., William Hucker F.J., Januzzi J.L., Thomas Ortel F.L., Saxonhouse S.J., Spinler S.A. (2017). 2017 ACC Expert Consensus Decision Pathway for Periprocedural Management of Anticoagulation in Patients with Nonvalvular Atrial Fibrillation: A Report of the American College of Cardiology Clinical Expert Consensus Document Task Force. J. Am. Coll. Cardiol..

[52] Mullins B., Akehurst H., Slattery D., Chesser T. (2018). Should surgery be delayed in patients taking direct oral anticoagulants who suffer a hip fracture? A retrospective, case-controlled observational study at a UK major trauma centre. BMJ Open.

[53] Vio R., Proietti R., Rigato M., Calò L.A. (2021). Clinical Evidence for the Choice of the Direct Oral Anticoagulant in Patients with Atrial Fibrillation According to Creatinine Clearance. Pharmaceuticals.

[54] Vanderwerf J.D., Kumar M.A. (2017). Management of neurologic complications of coagulopathies. Handb. Clin. Neurol..

[55] Yassa R., Khalfaoui M.Y., Hujazi I., Sevenoaks H., Dunkow P. (2017). Management of anticoagulation in hip fractures: A pragmatic approach. EFORT Open Rev..

[56] Kietaibl S., Ferrandis R., Godier A., Llau J., Lobo C., Macfarlane A.J., Schlimp C.J., Vandermeulen E., Volk T., Von Heymann C. (2022). Regional anaesthesia in patients on antithrombotic drugs Joint ESAIC/ESRA guidelines. Eur. J. Anaesthesiol..

[57] Gulcelik N.E., Bayraktar M., Caglar O., Alpaslan M., Karakaya J. (2011). Mortality after hip fracture in diabetic patients. Exp. Clin. Endocrinol. Diabetes.

[58] Chen Y., Yang X., Meng K., Zeng Z., Ma B., Liu X., Qi B., Cui S., Cao P., Yang Y. (2013). Stress-Induced Hyperglycemia After Hip Fracture and the Increased Risk of Acute Myocardial Infarction in Nondiabetic Patients. Diabetes Care.

[59] Grintescu I.M., Luca Vasiliu I., Cucereanu Badica I., Mirea L., Pavelescu D., Balanescu A., Grintescu I.C. (2015). The influence of parenteral glutamine supplementation on glucose homeostasis in critically ill polytrauma patients—A randomized-controlled clinical study. Clin. Nutr..

[60] Allan B. Management of Adults with Diabetes Undergoing Surgery and Elective Procedures: Improving Standards Summary Supporting Organisations JBDS IP Group with Special Thanks to Christine Jones (Norwich) for Her Administrative Work and Help with These Guidelines and with JBDS-IP n.d. https://abcd.care/sites/default/files/site_uploads/JBDS_Guidelines_Archive/JBDS_03_Surgical_guidelines_2015_full_FINAL_amended_Mar_2016_Archive.pdf.

[61] Lo I.L., Siu C.W., Tse H.F., Lau T.W., Leung F., Wong M. (2010). Pre-operative pulmonary assessment for patients with hip fracture. Osteoporos. Int..

[62] Loggers S.A.I., Giannakopoulos G.F., Vandewalle E., Erwteman M., Berger F., Zuidema W.P. (2017). Preoperative chest radiographs in hip fracture patients: Is there any additional value?. Eur. J. Orthop. Surg. Traumatol..

[63] Patterson J.T., Bohl D.D., Basques B.A., Arzeno A.H., Grauer J.N. (2017). Does Preoperative Pneumonia Affect Complications of Geriatric Hip Fracture Surgery?. Am. J. Orthop..

[64] Jamali S., Dagher M., Bilani N., Mailhac A., Habbal M., Zeineldine S., Tamim H. (2018). The Effect of Preoperative Pneumonia on Postsurgical Mortality and Morbidity: A NSQIP Analysis. World J. Surg..

[65] Badrick T., Turner P. (2013). The Uncertainty of the eGFR. Indian J. Clin. Biochem..

[66] Ostermann M., Zarbock A., Goldstein S., Kashani K., Macedo E., Murugan R., Bell M., Forni L., Guzzi L., Joannidis M. (2020). Recommendations on Acute Kidney Injury Biomarkers from the Acute Disease Quality Initiative Consensus Conference A Consensus Statement. JAMA Netw. Open.

[67] Marakala V. (2022). Neutrophil gelatinase-associated lipocalin (NGAL) in kidney injury—A systematic review. Clin. Chim. Acta.

[68] Hong S.E., Kim T.Y., Yoo J.H., Kim J.K., Kim S.G., Kim H.J., Song Y.R. (2017). Acute kidney injury can predict in-hospital and long-term mortality in elderly patients undergoing hip fracture surgery. PLoS ONE.

[69] Graver A., Merwin S., Collins L., Kohn N., Goldman A. (2015). Comorbid Profile Rather than Age Determines Hip Fracture Mortality in a Nonagenarian Population. HSS J.^®^.

[70] Juthani-Mehta M. (2007). Asymptomatic Bacteriuria and Urinary Tract Infection in Older Adults. Clin. Geriatr. Med..

[71] Bliemel C., Buecking B., Hack J., Aigner R., Eschbach D.A., Ruchholtz S., Oberkircher L. (2017). Urinary tract infection in patients with hip fracture: An underestimated event?. Geriatr. Gerontol. Int..

[72] Gillespie W.J., Walenkamp G.H. (2010). Antibiotic prophylaxis for surgery for proximal femoral and other closed long bone fractures. Cochrane Database Syst. Rev..

[73] Bonaccorsi H.A., Burns B. (2024). Perioperative Cardiac Management.

[74] Whinney C. (2009). Perioperative medication management: General principles and practical applications. Clevel. Clin. J. Med..

[75] Freter S., Dunbar M., Koller K., MacKnight C., Rockwood K. (2016). Prevalence and Characteristics of Pre-Operative Delirium in Hip Fracture Patients. Gerontology.

[76] Cotae A.M., Mirea L., Cobilinschi C., Ungureanu R., Grintescu I.M. (2024). Early postoperative cognitive decline—Are there any preventive strategies for surgical patients in the emergency setting?. Signa Vitae.

[77] Kim S.Y., Jo H.Y., Na H.S., Han S.H., Do S.H., Shin H.J. (2023). The Effect of Peripheral Nerve Block on Postoperative Delirium in Older Adults Undergoing Hip Surgery: A Systematic Review and Meta-Analysis of Randomized Controlled Trials. J. Clin. Med..

[78] Al-Husinat L., Jouryyeh B., Al Sharie S., Al Modanat Z., Jurieh A., Al Hseinat L., Varrassi G. (2023). Bone Cement and Its Anesthetic Complications: A Narrative Review. J. Clin. Med..

[79] Moldovan F. (2023). Bone Cement Implantation Syndrome: A Rare Disaster Following Cemented Hip Arthroplasties—Clinical Considerations Supported by Case Studies. J. Pers. Med..

[80] Popescu D., Ene R., Popescu A., Cîrstoiu M., Sinescu R., Cîrstoiu C. (2015). ToTal hip joinT replacemenT in young male paTienT wiTh osTeoporosis, secondary To hypogonadoTropic hypogonadism. Acta Endocrinol..

[81] El-Othmani M.M., Zalikha A.K., Cooper H.J., Shah R.P. (2022). Femoral Stem Cementation in Primary Total Hip Arthroplasty. JBJS Rev..

